# Cerebral Embolic Protection During Transseptal Biopsy of Left Atrial Mass

**DOI:** 10.1016/j.jaccas.2022.05.020

**Published:** 2022-08-03

**Authors:** Solomon A. Seifu, Michael Fabbro, Mauricio G. Cohen

**Affiliations:** Cardiovascular Division, Department of Medicine, University of Miami Miller School of Medicine, Miami, Florida, USA

**Keywords:** cardiac magnetic resonance, cardiovascular disease, cardiac tomography, imaging, left-sided catheterization, nuclear medicine, palpitations, tachycardia, treatment, FDG, fluorodeoxyglucose, PET, positron emission tomography, TEE, transesophageal echocardiography, TTE, transthoracic echocardiography

## Abstract

Diagnostic biopsy of a left atrial mass is technically feasible but has the risk of tumor embolization causing stroke or seeding. In this case report, we highlight the technical steps for left atrial mass biopsy under transesophageal echocardiography guidance by using cerebral embolic protection. Pathologic examination disclosed low-grade B-cell lymphoma. (**Level of Difficulty: Advanced.**)

## Central Illustration

**Figure:**
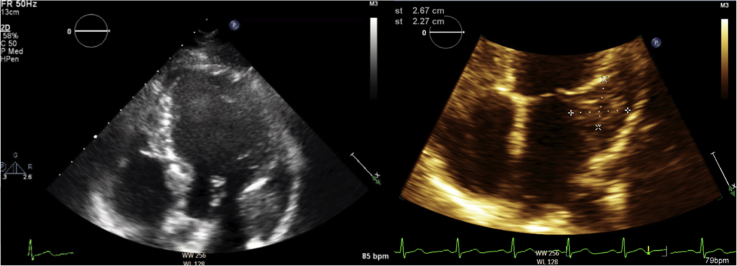

## History of Presentation

A 52-year-old woman initially presented to her primary care physician with reports of fatigue, chest discomfort, and intermittent palpitations. She noticed multiple brief episodes of tachycardia, with her heart rate rising to approximately 130 beats/min. She denied associated dizziness or syncope. Transthoracic echocardiography (TTE) revealed a mass in the left atrium, for which she was referred to a cardiologist. Her vital signs were stable, with blood pressure of 125/85 mm Hg, and she was afebrile and not tachycardic. Physical examination showed normal heart sounds with no murmur, as well as good air entry in both lungs, absence of lower extremity edema, and no palpable masses or lymph nodes. She denied unintended weight loss or other constitutional symptoms.

**Learning Objectives**

• To understand the significance of various cardiac imaging modalities for planning and guidance of percutaneous left atrial mass biopsy procedure.
• To learn the steps, safety, and importance of percutaneous transseptal left atrial biopsy.
• To highlight the importance of using a cerebral embolic protection device during percutaneous left atrial mass biopsy.

## Past Medical History

The patient had a history of COVID-19 about a year before presentation. She also had history of gastroesophageal reflux, for which she took pantoprazole.

## Investigations

**Figure 1:**
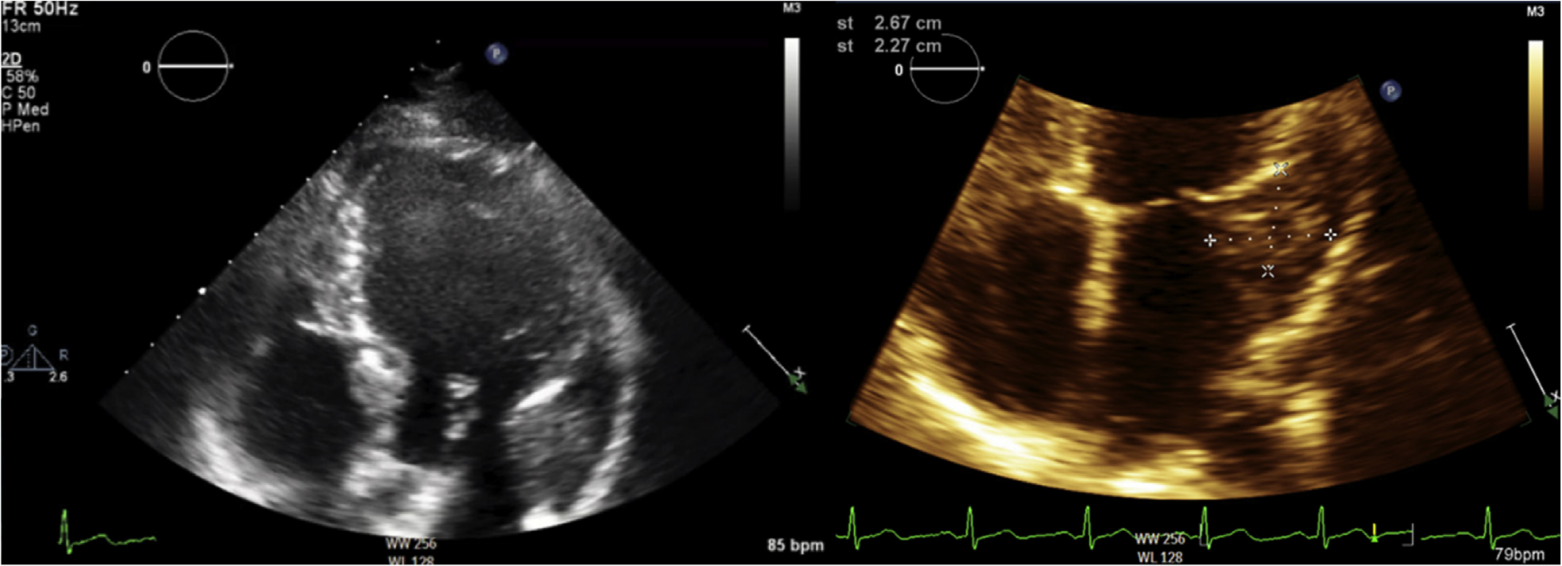
Transthoracic Echocardiography With the Mass on the Lateral Left Atrial Wall

**Figure 2:**
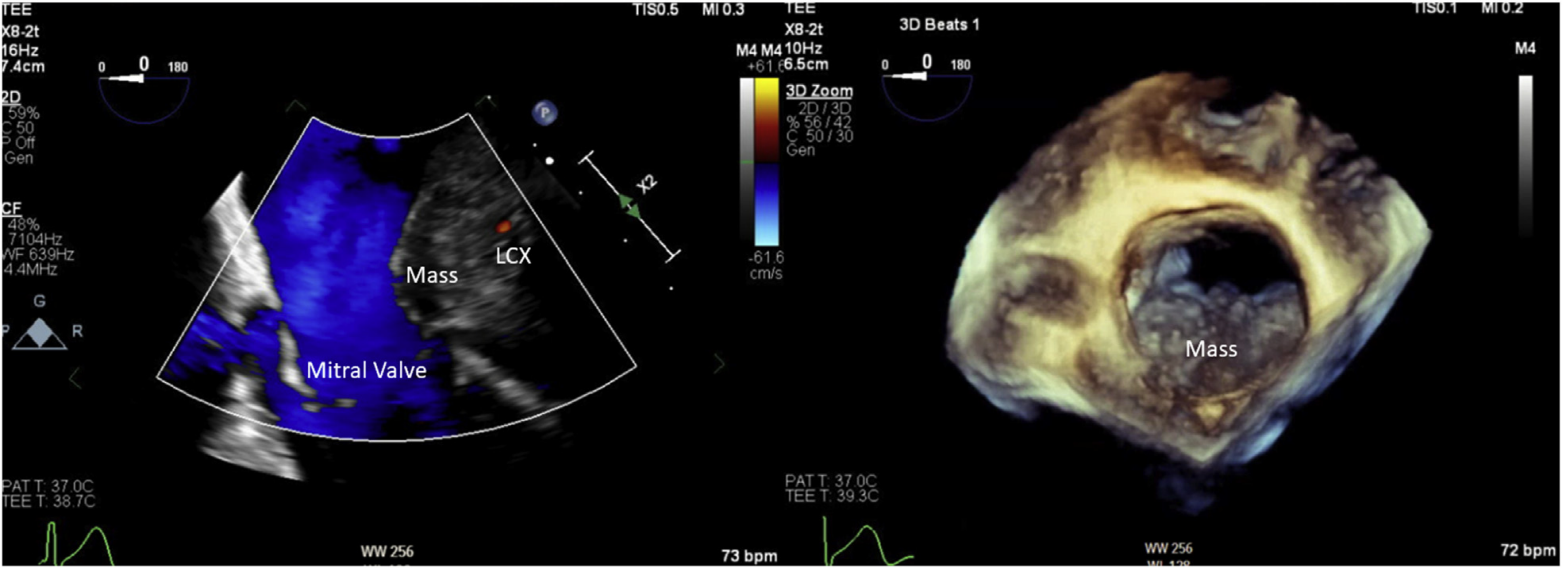
Transesophageal Echocardiography Showing the Mass in the Lateral Left Atrial Wall and Atrioventricular Groove Encasing the Left Circumflex Artery LCX = left circumflex.

**Figure 3:**
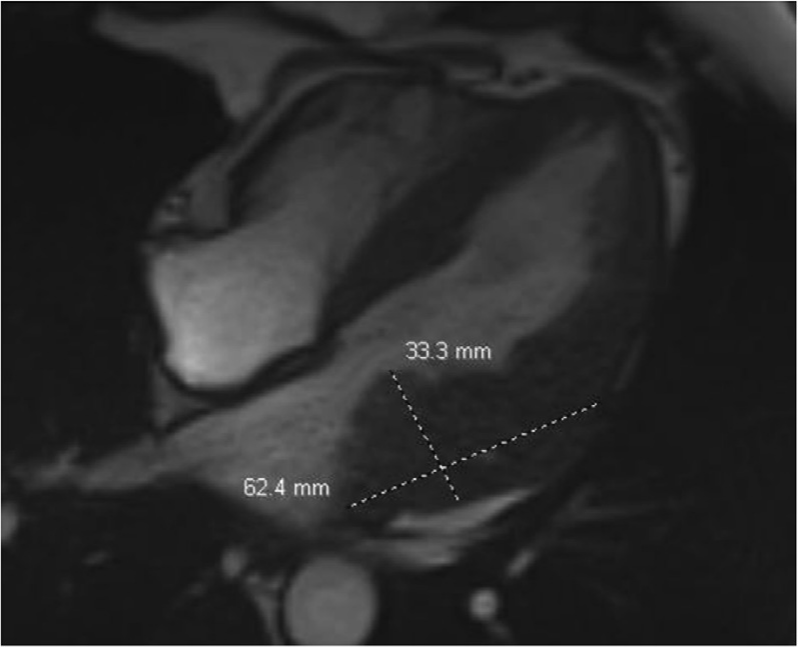
Cardiac Magnetic Resonance Showing the Mass

**Figure 4:**
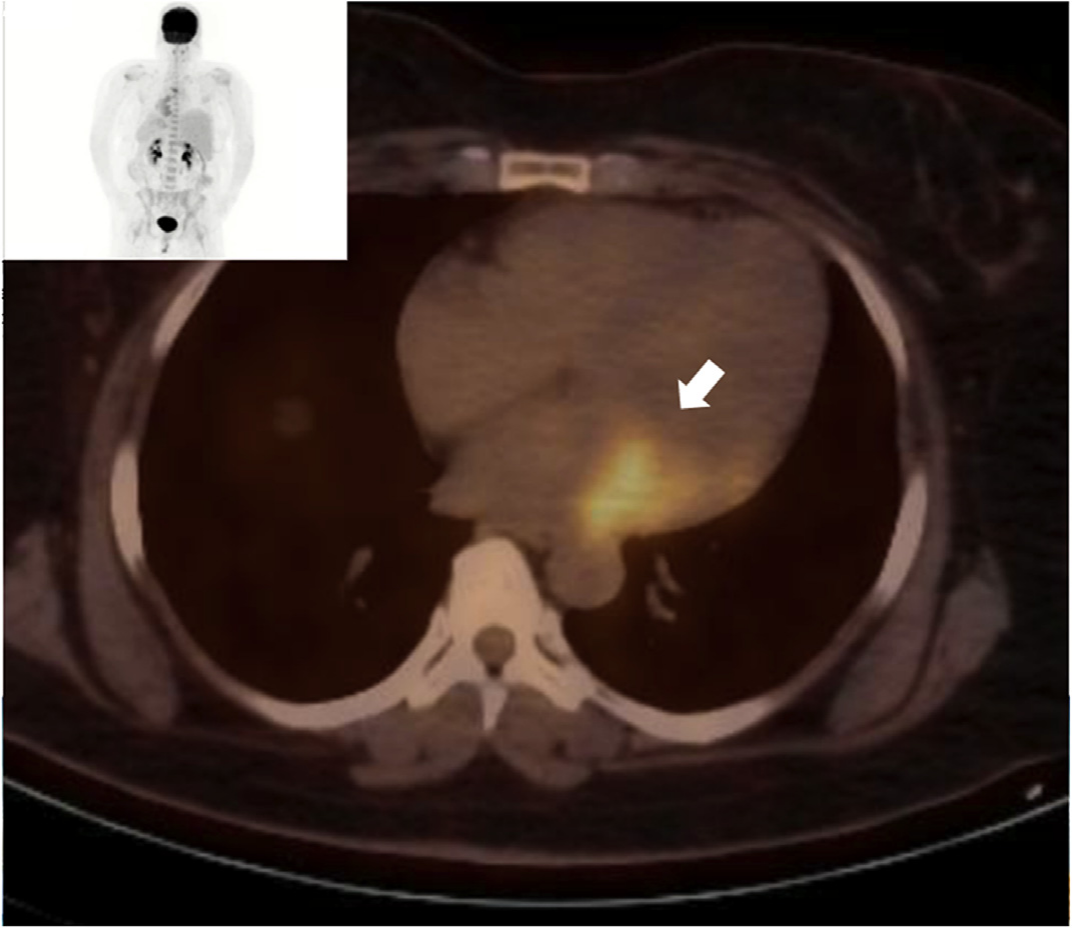
Positron Emission Tomography With Fluorodeoxyglucose Uptake Over the Cardiac Mass The **arrow** points to the fluorodeoxyglucose uptake area.

**Figure 5:**
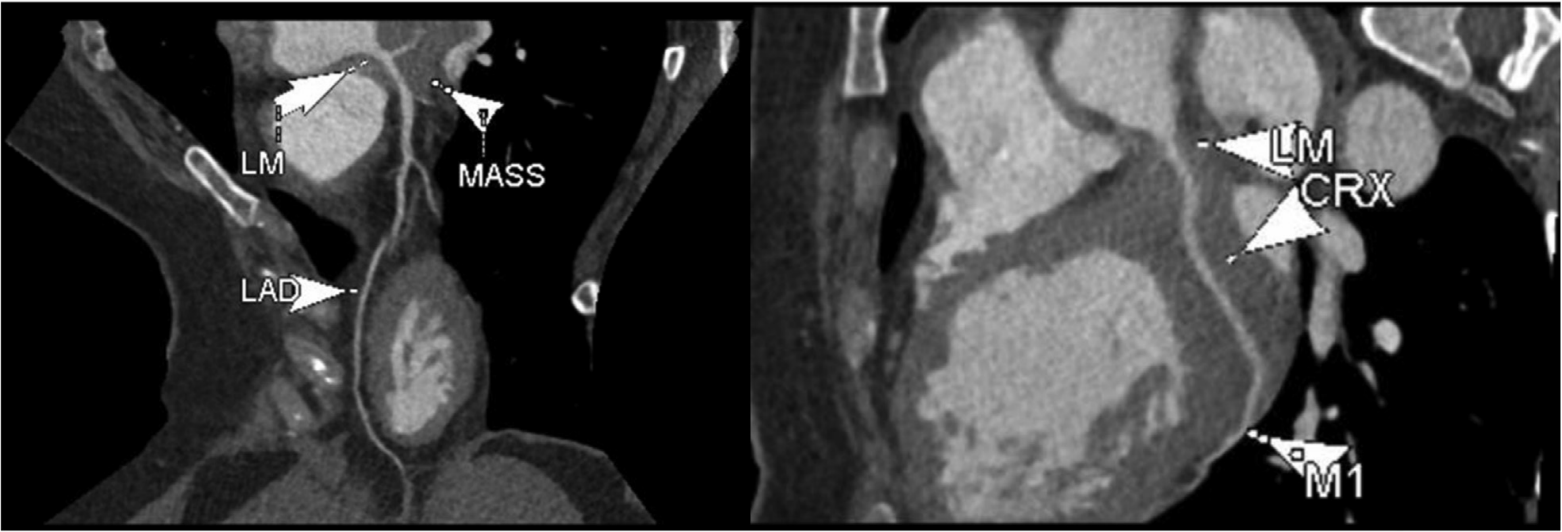
Cardiac Computed Tomography With Oblique Multiplanar Reconstruction The image shows the tumor encasing the left main (LM) and proximal left anterior descending (LAD) coronary arteries. CRX = left circumflex artery; M1 = first obtuse marginal artery.

Laboratory data demonstrated normal cell counts with normal renal and liver function. The electrocardiogram showed normal sinus rhythm. TTE showed a large mass in the left atrium attached to the lateral wall that was compatible with tumor (Figure 1). Transesophageal echocardiography (TEE) showed the large mass attached to the lateral wall of the left atrium extending toward the left ventricle and encasing the posterior mitral valve leaflet as well as the left circumflex artery, with no involvement of the left atrial appendage and the left upper pulmonary vein (Figure 2, Video 1). Cardiac magnetic resonance showed a 6.5 × 3.3 cm mass that was inseparable from the myocardium and left atrial wall. This mass infiltrated mostly the basal lateral wall and the anterior and inferior basal wall of the myocardium (Figure 3). A whole-body positron emission tomography (PET) scan showed a fluorodeoxyglucose (FDG)–avid lesion in the basal left ventricular myocardium and the lateral aspect of the left atrium (Figure 4). A multislice computed tomography cardiac morphology scan showed a diffusely infiltrative mass encasing the left main, left circumflex, and proximal left anterior descending coronary arteries (Figure 5). A right ventricular biopsy showed normal cardiac tissue with mild myocyte hypertrophy and patchy mild interstitial fibrosis.

## Differential Diagnosis

Cardiac tumors can be broadly categorized into neoplastic and non-neoplastic. Neoplastic tumors are further categorized into primary cardiac tumors and secondary metastatic cardiac tumors. In our case, given the diffuse infiltrative nature of the mass and absence of tumor in any other part of the body, a primary cardiac malignant tumor was more likely than a secondary metastatic tumor. The differential diagnosis included angiosarcoma, rhabdomyosarcoma, leiomyosarcoma, liposarcoma, osteosarcoma, fibrosarcoma, and lymphoma.

## Management

**Figure 6:**
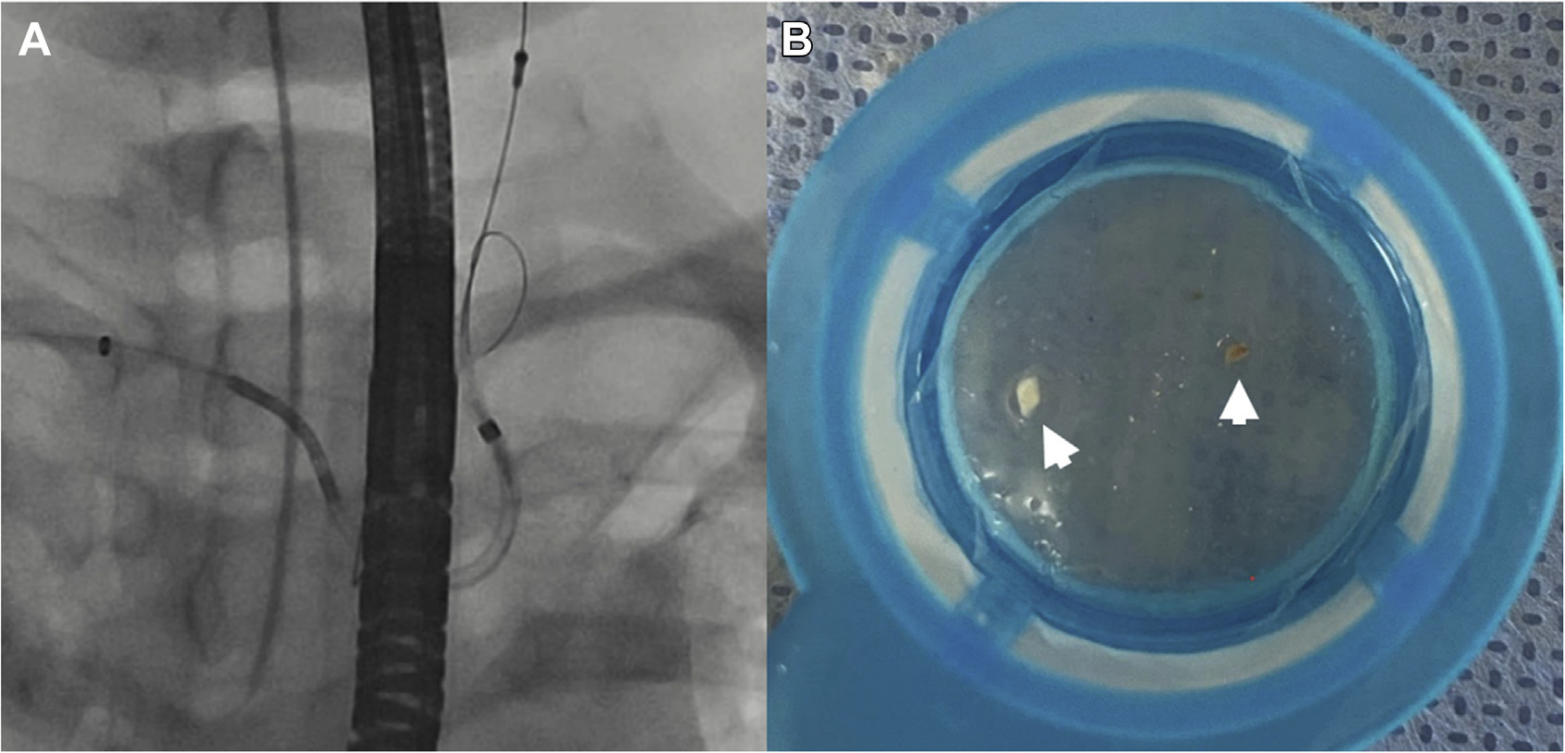
Cerebral Embolic Protection Device Deployment and Debris Retrieved **(A)** Cerebral embolic protection device deployed in the brachiocephalic and left common carotid arteries. **(B)** Debris **(arrows)** retrieved from the cerebral protection device.

**Figure 7:**
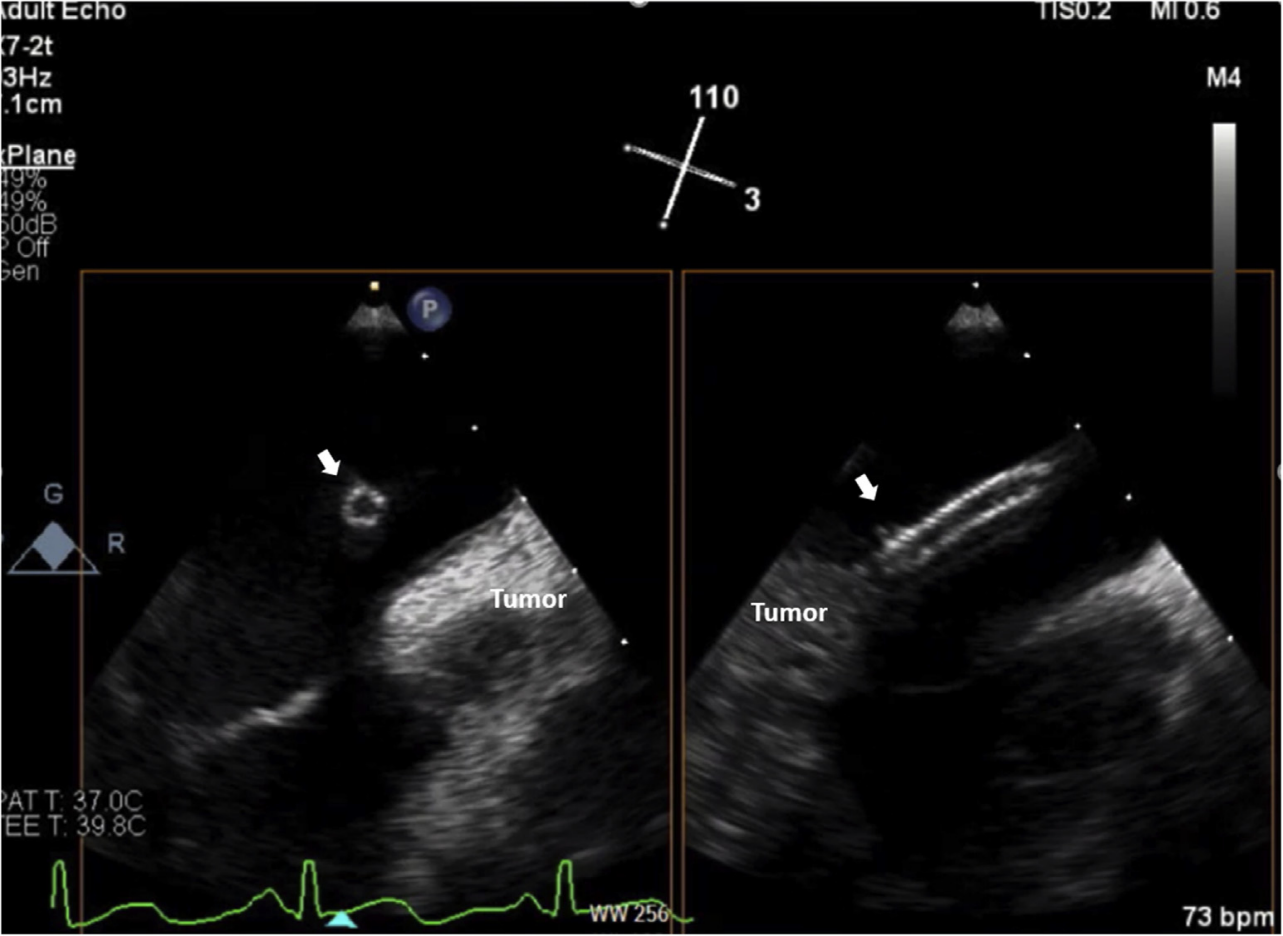
Transesophageal Echocardiography With Biplane Imaging During Endomyocardial Biopsy The image shows the biopsy forceps **(arrows)** directed against the cardiac mass and sampling the mass.

**Figure 8:**
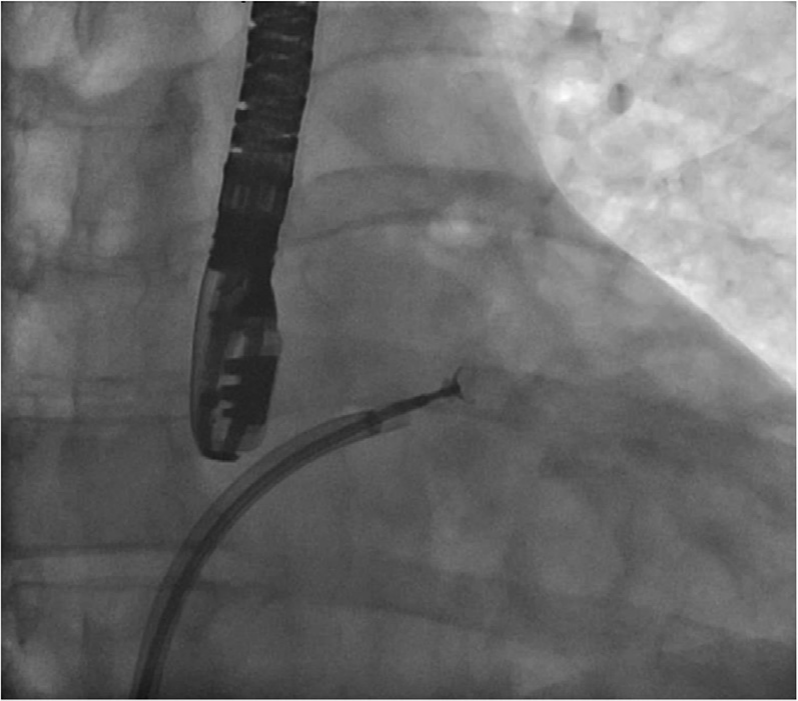
Fluoroscopic Image With Open Arms of the Endomyocardial Biopsy Forceps Sampling the Left Atrial Mass

**Figure 9:**
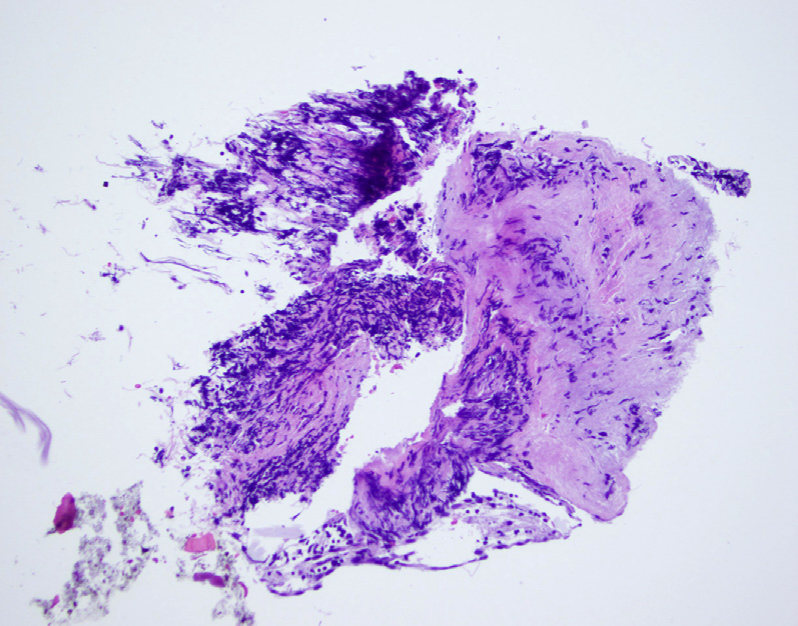
Cardiac Mass Histopathology Showing Infiltration With Atypical Lymphoid Cells

The patient’s case was discussed by a multidisciplinary team, and the decision was made to biopsy the left atrial mass through a percutaneous transseptal approach. Use of cerebral embolic protection was planned to avoid the risk of stroke caused by tumor embolization. The procedure was performed with the patient under general anesthesia with fluoroscopic and TEE guidance. A cerebral embolic protection device (Boston Scientific) was delivered through right radial access, and the filters were deployed in the right brachiocephalic and left common carotid arteries (Figure 6A). Through right femoral venous access, we advanced an 8-F transseptal guiding sheath (Baylis) and punctured the interatrial septum at a midlocation in the fossa ovalis, by using radiofrequency energy delivered through a radiofrequency wire (Baylis). The transseptal sheath was exchanged for an 8.5-F steerable Agilis guide catheter (Abbott Vascular). Under 2- and 3-dimensional TEE guidance, the steerable sheath was directed toward the left atrial mass (Figure 7), and 6-F endomyocardial biopsy forceps (Argon Medical) were advanced towards the mass (Figure 8). A total of 6 samples were taken from different areas of the left atrial mass (Videos 2 and 3). At the end of the procedure, the cerebral embolic protection device was removed, and captured debris was retrieved (Figure 6B). The biopsy samples together with the debris retrieved from the embolic protection device were sent for pathologic examination, which revealed low-grade B-cell lymphoma (Figure 9).

## Discussion

Cardiac tumors are rare but constitute an important challenge with respect to diagnosis because they may not be easily accessible for biopsy through a percutaneous approach. Primary cardiac tumors are extremely rare, with an incidence of <0.1% as compared with secondary metastatic cardiac tumors, which are more than 20 times more common.^1^ Tumors that look removable from a noninvasive imaging study are often surgically resected, but tumors that appear diffuse or unresectable pose a particular challenge. The pathologic features of the tumors are crucial in guiding subsequent nonsurgical treatment. The use of percutaneous myocardial biopsy is well established in the investigation of cardiomyopathies, monitoring of transplant rejection, and diagnosis of diffuse endocardial disease because tissue samples are usually obtained from the right ventricle. However, biopsy of localized masses in the left side of the heart is technically challenging and requires planning and imaging guidance The use of intracardiac echocardiography for biopsy of a right-sided cardiac mass has been described in few previous case reports.^2^ Percutaneous left atrial mass biopsy has an associated procedural risk of tumor embolization. The most serious complications of such embolization are neurologic.^3^ Even though the clinical usefulness of cerebral embolic protection devices is uncertain, we believed that maneuvering catheters in the left atrium with an already existing tumor would undoubtedly increase the risk of embolization and thus limit the utility of the transseptal approach. The percutaneous transseptal approach with 3-dimensional TEE guidance has been described in the past,^4^ but our case is the first to illustrate the feasibility and potential benefit of a cerebral embolic protection strategy during percutaneous transseptal biopsy of left-sided cardiac masses. Small pieces of debris were retrieved from the cerebral embolic protection device in our patient. Our case further supports the safety of the percutaneous transseptal approach under TEE guidance of biopsy forceps through a deflectable sheath in the left atrium toward the tumor. Pathologic study of the biopsy specimen from our patient showed low-grade B-cell lymphoma, a primary cardiac lymphoma.^5^ Most primary cardiac lymphomas either are diagnosed at autopsy or are fatal soon after diagnosis. In our case, with a less invasive percutaneous transseptal approach, the diagnosis was safely made, and our patient was referred to oncology for early institution of chemotherapy.

## Follow-Up

**Figure 10:**
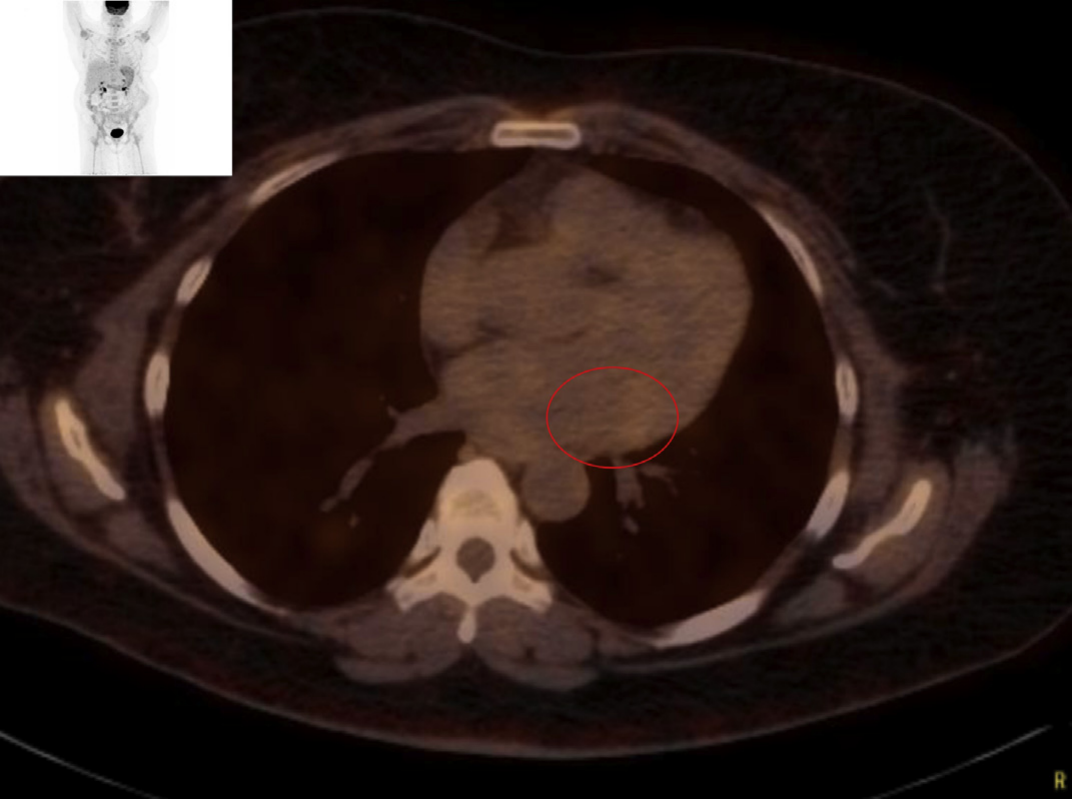
Positron Emission Tomography Scan With Resolution of the Cardiac Mass The **red circle** shows the area of resolution of fluorodeoxyglucose uptake.

Bone marrow biopsy showed no involvement. After completion of 6 cycles of chemotherapy with bendamustine and rituximab, a PET scan showed resolution of the previously noted hypermetabolic left atrial mass (Figure 10).

## Conclusions

Compared with the traditional surgical approach, percutaneous transseptal left arial biopsy with cerebral embolic protection is safe and less invasive, especially for patients who are not candidates for surgical resection. With the advancement of structural heart interventional techniques, as well as refinement of cardiac imaging modalities, percutaneous transseptal biopsy is a reliable and viable option for sampling left atrial masses. A cerebral embolic protection strategy may reduce the risk of stroke potentially associated with this procedure.

## Funding Support and Author Disclosures

The authors have reported that they have no relationships relevant to the contents of this paper to disclose.

## Appendix

Supplemental Video 1 TEE Showing the Left Atrial Mass

Supplemental Video 2 TEE Image Showing Biopsy Sampling From the Left Atrial Mass

Supplemental Video 3 Endomyocardial Biopsy Forceps Sampling the Left Atrial Mass
